# Hubbing

**Published:** 1857-12

**Authors:** 


### "HUB ME, SHIPMATE."

VERY many pleasant letters *and dollars!* have come to us from the North, South, East, and West, in consequence of our October article under the above heading.

*North Carolina* says: "Please find inclosed for the four bound volumes of your Journal, for my friend. Thus much do I 'Hub' you, and hope to be able to do so again. O. S. B."

*New York City:* "While reading the *Home Journal* for this week, which, I am happy to say, I have an opportunity of doing weekly, I came to an article headed, 'Hub me, Shipmate,' taken from your useful Journal. This put me in mind of what I intended to do for a long time, that is, to subscribe for your Journal; so that the said *Home Journal* has 'hubbed' me on your account very effectually. This article struck me so forcibly, that although intent on reading, I determined to leave the paper immediately, to order your Journal. A. N."

*Massachusetts:* "I have read all the numbers I ordered, and want more: I have been more interested than I expected to be. The commendations of your Journal are high, but not higher than it merits. I have long been wishing for such a publication: I became interested in it by seeing, within a year past, somewhat frequent, and always valuable quotations from it in the *Congregational* of Boston. I propose to send away the numbers for this year, and order the bound volume. C. M. C."

*Wilmington:* "The first article which arrested my attention to-day, on opening the JOURNAL OF HEALTH, was '*Hub me, Shipmate.*' I read it to an intelligent gentleman, in my office at the time, and 'hubbed' the Journal by a word of approbation and solicitation, and the inclosed is the result. Accept this as another instance of 'Hubbing,' from one who values the monthly visits of the Journal highly, because it brings both profit and pleasure to your friend and obedient servant.

W. G. T."

It is natural for us, on receiving any good, to desire to impart it to others, and thus double its benefits. In proportion, then, as the reader has been entertained, instructed, and benefited by perusing what we have written during the past year, may we not hope that, by dropping a word of approbation of us here and there, and following that word by a solicitation to subscribe, a good may be done all round, and many new subscribers come in to us.

